# Supplementary material for: Diversification of dentate gyrus granule cell subtypes is regulated by Nrg1 nuclear back-signaling
Source: Life Sci Alliance. 2025 Apr 25;8(7):e202403169. doi: 10.26508/lsa.202403169 (PMC12032840; doi:10.26508/lsa.202403169)
Supplement: Supplementary file 7 [file LSA-2024-03169_Supplemental_Data_1.docx]

**Key Resources Table**

| **REAGENT or RESOURCE** | **SOURCE** | **IDENTIFIER** |
| --- | --- | --- |
| **Fluorescent Detection Reagents** | | |
| Streptavidin, Alexa Fluor™ 647 conjugate  (1:1000) | ThermoFisher | S21374 |
| Opal dye 520 | Akoya Biosciences | FP1487001KT |
| Opal dye 570 | Akoya Biosciences | FP1488001KT |
| Opal dye 620 | Akoya Biosciences | FP1495001KT |
| **Chemicals, Peptides, and Recombinant Proteins** | | |
| DAPI Fluoromount-G® | SouthernBiotech | Cat# 0100-20 |
| NEUROBIOTIN® Tracer | Vector Laboratories | Cat# SP-1120 |
| Potassium gluconate | Sigma-Aldrich | Cat# P1847 |
| Potassium chloride | Sigma-Aldrich | Cat# P4504 |
| HEPES | Sigma-Aldrich | Cat# H3375 |
| Magnesium chloride hexahydrate | Sigma-Aldrich | Cat# M2393 |
| Adenosine 5′-triphosphate magnesium salt | Sigma-Aldrich | Cat# A9187 |
| Guanosine 5′-triphosphate sodium salt hydrate | Sigma-Aldrich | Cat# G8877 |
| Phosphocreatine disodium salt hydrate | Sigma-Aldrich | Cat# P7936 |
| Sodium bicarbonate | Sigma-Aldrich | Cat# S6014 |
| D-(+)-Glucose | Sigma-Aldrich | Cat# G7528 |
| Sodium phosphate monobasic monohydrate | Sigma-Aldrich | Cat# S9638 |
| Calcium chloride dihydrate | Sigma-Aldrich | Cat# C7902 |
| Magnesium sulfate | Sigma-Aldrich | Cat# M7506 |
| Sucrose | Sigma-Aldrich | Cat# S9378 |
| AnaSed® (xylazine injection) | Akorn | Cat# 59399-110-20 |
| ZETAMINE- ketamine hydrochloride injection | VetOne | Cat# 13985-584-10 |
| Forane Inhalation Solution 99.9% | Henry Schein | Cat# 1100734 |
| **Critical Commercial Assays** | | |
| Chromium Nuclei Isolation with RNase Inhibitor Kit, 16rxns | 10x Genomics | Cat# 1000494 |
| Chromium Next GEM Single Cell Multiome ATAC + Gene Expression Reagent Bundle | 10x Genomics | Cat# 1000283 |
| RNAScope™ Fluorescent Multiplex v2 Kit | ACD | 323136 |
| RNAscope® 4-Plex Ancillary kit | ACD | 323120 |
| RNAscope™ Intro Pack for HiPlex12 Reagents Kit - Mm v2 | ACD | 324440 |
| **Deposited** **Data** | | |
| snMultiome raw & processed data | This study | GEO Accession number: GSE280167 |
| **Experimental Models: Organisms/Strains** | | |
| Mouse: Nrg1^tm1DatV/L^ | Dr. David A. Talmage, NINDS | Jax Labs Stock # 040044 |
| **Oligonucleotides** | | |
| Genotyping Fwd Primer:  5'-GGTGATCCCATACCCAAGACTCAG-3' | IDT |  |
| Genotyping Rev Primer:  5'-CTGCACATTTATAGAGCATTTATTTTG -3' | IDT |  |
| RNAscope® HiPlex Probe - Mm-Penk-T1 – Mus musculus preproenkephalin (Penk), mRNA | ACD | 318761-T1 |
| RNAscope® HiPlex Probe - Mm-Nptx2-T2 – Mus musculus neuronal pentraxin 2 (Nptx2), mRNA | ACD | 316901-T2 |
| RNAscope® HiPlex Probe - Mm-Sorcs3-T7- musculus sortilin-related VPS10 domain  containing receptor 3 (Sorcs3), mRNA | ACD | 473421-T7 |
| RNAscope® HiPlex Probe - Mm-Vgf-T5 – Mus musculus VGF nerve growth factor inducible (Vgf), mRNA | ACD | 517421-T5 |
| RNAscope® HiPlex Probe - Mm-Cit-T4 – Mus musculus citron (Cit), mRNA | ACD | 567401-T4 |
| RNAscope® HiPlex Probe - Mm-Scg2-T3 -Mus musculus secretogranin II (Scg2) transcript variant 1, mRNA | ACD | 477691-T3 |
| RNAscope® HiPlex Probe - Mm-Itgav-T10 – Mus musculus integrin alpha V (Itgav), mRNA | ACD | 513901-T10 |
| RNAscope® HiPlex Probe - Mm-Gpnmb-T11 -Mus musculus glycoprotein (transmembrane) nmb  (Gpnmb), mRNA | ACD | 489511-T11 |
| RNAscope® HiPlex Probe - Mm-Meg3-T7 -Mus musculus maternally expressed 3 (Meg3)  transcript variant 1 long non-coding RNA | ACD | 527201-T7 |
| RNAscope® Probe - Mm-Nrg1-O5-C3 Mus musculus neuregulin 1 (Nrg1) transcript variant 3,  mRNA | ACD | 870451-C3 |
| RNAscope® Probe - Mm-Dcx-C2 – Mus musculus doublecortin (Dcx) transcript variant 1, mRNA | ACD | 478671-C2 |
| RNAscope® Probe - Mm-Penk - Mus musculus preproenkephalin (Penk), mRNA | ACD | 318761 |
| RNAscope® Probe - Mm-Camk4-C3 – Mus musculus calcium/calmodulin-dependent protein kinase IV (Camk4), mRNA | ACD | 586771-C3 |
| RNAscope® Probe - Mm-Ntng1-C4 – Mus musculus netrin G1 (Ntng1) transcript variant a, mRNA | ACD | 488871-C4 |
| RNAscope® Probe - Mm-Nptx2-C4 – Mus musculus neuronal pentraxin 2 (Nptx2), mRNA | ACD | 316901-C4 |
| RNAscope® Probe - Mm-Sorcs3-C2 – Mus musculus sortilin-related VPS10 domain containing receptor  3 (Sorcs3), mRNA | ACD | 473421-C2 |
| RNAscope™ Probe - Mm-Nrg1-O6-C4,Mus musculus neuregulin 1 (Nrg1) transcript variant 1, mRNA | ACD | 1568461-C4 |
| **Software and Algorithms** | | |
| R Code for snRNA+ATAC-Seq analysis | This study | https://github.com/RajNINDS/V321L-snMultiome |
| Code for electrophysiology data analysis | This study | https://github.com/nsdesai/patch_clamp_analysis |
